# A comparison of antibody response in kidney transplant recipients and healthcare workers who had PCR-confirmed COVID-19 infection

**DOI:** 10.55730/1300-0144.5520

**Published:** 2022-09-12

**Authors:** Mevlüt Tamer DİNCER, Necmi EREN, Ahmet MURT, Nuriye YILDIZ, Şeyda Gül ÖZCAN, Metin ERGÜL, Sibel GÖKÇAY BEK, Zeynep ATLI, Sinan TRABULUS, Erkan DERVİŞOĞLU, Hamdi Levent DOĞANAY, Nurhan SEYAHİ

**Affiliations:** 1 Division of Nephrology, Department of Internal Medicine, Faculty of Medicine, İstanbul University-Cerrahpaşa, İstanbul, Turkey; 2 Division of Nephrology, Department of Internal Medicine, Faculty of Medicine, Kocaeli University, Kocaeli, Turkey; 3 Department of Internal Medicine, Faculty of Medicine, İstanbul University-Cerrahpaşa, İstanbul, Turkey; 4 Department of Account and Tax Application, Sinop University, Sinop, Turkey; 5 Genomic Laboratory (GLAB), Ümraniye Teaching and Research Hospital, University of Health Sciences, İstanbul, Turkey

**Keywords:** COVID-19, immune response, immunosuppression, kidney transplantation, SARS-CoV-2 IgG antibody

## Abstract

**Background/aim:**

Data on antibody response following COVID-19 in kidney transplant recipients is scarce. This crosssectional study aims to investigate the antibody response to COVID-19 among kidney transplant recipients.

**Materials and methods:**

We recruited 46 kidney transplant recipients with RT-PCR-confirmed COVID-19 and 45 recipients without COVID-19 history. We also constructed two control groups (COVID-19 positive and negative) from a historical cohort of healthcare workers. We used age and sex-based propensity score matching to select the eligible subjects to the control groups. We measured the SARS-CoV-2 IgG levels quantitatively using the Abbott ARCHITECT system. An antibody level above 1.4 S/C was defined as positivity.

**Results:**

Transplant recipients with COVID-19 had a higher BMI, and COVID-19 history in a household member was more common than that of the transplant recipient without COVID-19. IgG seropositivity rate (69.6% vs. 78.3%, p = 0.238) and the median IgG level (3.28 [IQR: 0.80–5.85] vs. 4.59 [IQR: 1.61–6.06], p = 0.499) were similar in COVID-19-positive transplant recipients and controls. Kidney transplant recipients who had a longer duration between RT-PCR and antibody testing had lower antibody levels (r = −0.532, p < 0.001).

**Conclusion:**

At the early post-COVID-19 period, kidney transplant recipients have a similar antibody response to controls. However, these patients’ antibody levels and immunity should be closely monitored in the long term.

## 1. Introduction

The coronavirus disease 2019 (COVID-19) pandemic caused by the SARS-CoV-2 virus has affected the global health system in an unprecedented way. The rapid development of various vaccines has become an important milestone in combating the pandemic. Vaccination relies on the production of antibodies via the stimulation of humoral immunity. However, data regarding antibody response to infection in immunosuppressed patients, including kidney transplant recipients, is not well defined.

Different mortality rates for COVID-19 were reported from different countries. According to a recent study in Western countries, mortality rates changed between 4.0% and 16.1%. Higher mortality rates were reported in patients with kidney disease in different studies, specifically in kidney transplantation patients having a mortality rate that ranges between 18.0% and 41.6% [1–6]. Moreover, data on the seropositivity rate following COVID-19 in kidney transplant recipients are scarce and not in agreement across studies. According to a recent study, lower antibody response rates (41.0%) were reported in kidney transplant recipients following recovery from the infection [7,8]. On the other hand, Azzi et al. investigated 69 kidney transplant recipients with reverse transcriptase-polymerase chain reaction (RT-PCR)-confirmed COVID-19 infection and found antinucleocapsid antibodies in 55 (80.0%) of them [9].

Usually, higher antibody response rates were reported for patients from the general population [8]. In a recent study, antinucleoprotein seropositivity rate was found 78.2% among healthcare workers with RT-PCR-confirmed COVID-19 [10].

In this crosssectional study, we aimed to investigate the prevalence of anti-SARS-CoV-2 antibodies in kidney transplant recipients first. Then, we examined the factors associated with the absence of antibody response.

## 2. Materials and methods

### 2.1. Study design

We performed a crosssectional study to recruit renal transplant recipients who were under regular follow-up at two university hospital’s transplantation centers (CMF and KMF) and had RT-PCR-confirmed COVID-19. We also recruited consecutive renal transplant recipients who did not have a history of COVID-19 and were attending the transplantation outpatient clinics in those institutions. We constructed two additional control groups from a previously screened cohort of healthcare workers [10]. None of the included patients was vaccinated for COVID-19 prior to the antibody measurement.

We used the descriptive comparative design method to assess the outcomes. The study protocol was approved by the Clinical Research Ethics Committee of İstanbul University-Cerrahpaşa (approval no: 2021-2921) and the Ministry of Health’s Scientific Committee (approval no: 2020-11-30T14_57_30). The study was conducted in accordance with the 1975 Declaration of Helsinki, as revised in 2013.

### 2.2. Sampling

The study was conducted between December 7, 2020, and February 12, 2021. The date of blood specimen collection for antibody measurement was accepted as the enrollment date to the study. The date of the RT-PCR testing was accepted as the first day of infection.

#### 2.2.1. Transplant recipients

Based on previous studies, we predicted the seropositivity following COVID-19 as 60% for transplant recipients [9,11] and 90% for subjects from the general population [12–14]. We performed a power analysis, and we planned to recruit 42 participants to each group.

A total of 623 patients had attended the outpatient transplantation clinics during the year 2020. Our target population comprised of patients who had COVID-19 following April, 2020. We did not formally screen all patients under follow-up; however, all of our transplant patients who had an RT-PCR-confirmed COVID-19 history were eligible for the study. We located 57 patients who had COVID-19, one of them died before the start of the study, 46 of them accepted to participate in the study.

We recruited transplant patients who gave informed consent in the COVID-19-negative group if they have not received a diagnosis of COVID-19 as of the recruitment day. We also checked if they had any RT-PCR test due to mandatory screening (before hospitalization due to any cause, having a household member with COVID-19) and confirmed that their RT-PCR test was negative. A total of 45 patients met the inclusion criteria for the control group.

Seventy-one of the 91 transplant recipients included in the study received a transplant from a living donor, while all donors were first- or second-degree relatives.

#### 2.2.2. Controls

We recruited control subjects from a cohort of healthcare workers that we examined previously [10]. The details of the recruitment and data collection for those participants were previously described in detail [10].

In that cohort, 116 subjects were RT-PCR positive. We excluded any subjects with malignancy or using immunosuppressive drugs. We transformed the duration between RT-PCR and antibody testing to binomial data based on a cutoff value of 8 weeks. The RT-PCR-positive control group is formed by recruiting subjects, using propensity score matching based on age, sex, and transformed antibody testing duration data with a 1:1 ratio.

Among healthcare workers who did not have a history of COVID-19, we selected the subjects designated as “no risk” (healthcare workers who were not attending the hospital because of administrative changes related to the pandemic) regarding COVID-19. We excluded any subjects with malignancy or using immunosuppressive drugs. Finally, 106 subjects were eligible for selection. We used age and sex-based propensity score matching to select subjects from this cohort with a ratio of 1:1.

We used the same laboratory procedures to measure the antibody levels in those subjects and transplant recipients.

### 2.3. Data collection

We filled in a standard form for every patient. We used patient interviews, medical records of the patients, the hospital’s electronic database, and the national public health data management system to collect data. Our form consisted of the following parts; demographics, clinical data including transplantation history, drug use, laboratory parameters, history, and clinical data related to COVID-19, and computed tomography (CT) findings. We also used the COVID-19 severity index to classify the patients under five mutually exclusive categories; asymptomatic or presymptomatic, mild, moderate, severe, critical illness [15].

### 2.4. PCR testing and assessment of antibodies

We used the same methods for RT-PCR testing and SARS-CoV-2- antibody measurement as described in detail previously [10,16]. For the detection of COVID-19 RNA, a commercial RT-PCR kit (Bio-Speedy SARS-CoV-2 RT-qPCR kit; Bioeksen R&D Technologies Ltd., İstanbul, Turkey) was used. For the detection of SARS-CoV-2 IgG (antinucleocapsid protein antibodies), chemiluminescent microparticle immunoassay (Abbott Laboratories, cat. no: 6R86, lot no: 16253FN00) was carried out according to the manufacturer’s instructions, and samples were run on the related instrument (ARCHITECT; Abbott Laboratories, Abbott Park, IL, USA). The qualitative results were reported by the instrument with a cutoff value of 1.40 S/C as recommended.

### 2.5. Therapeutic approach

Antiviral therapy with favipiravir and prophylactic anticoagulation with low-molecular-weight heparin were considered in all patients unless contraindicated. For patients with systemic inflammation, tocilizumab or anakinra was initiated in addition to high-dose corticosteroids. Regarding immunosuppressive treatment, antiproliferative agents were ceased or reduced, calcineurin inhibitors were maintained stable, dose-reduced or were completely withdrawn, and corticosteroid doses were increased based on the disease severity.

### 2.6. Statistical analysis

Descriptive data were presented as mean ± standard deviation (SD) and median and interquartile range (IQR) for the continuous variables and frequency and percentages (%) for the categorical variables. Continuous variables were evaluated for normality using the Shapiro-Wilk test. Kidney transplant recipients and control groups were compared with an independent samples t-test for normally distributed variables and the Mann-Whitney U test for nonnormally distributed variables. Categorical variables were compared using the chi-square or Fisher’s exact test for proportion. Multivariate analysis was applied to determine the association between the antibody level, the groups, and postinfection duration. All significance tests were two-tailed, and values of p <0.05 were considered statistically significant. All statistical analyses were performed using the SPSS software version 21 (IBM Corporation, Armonk, NY, USA).We employed propensity score matching to balance in the observed baseline covariates and reduce the bias of treatment effect between the kidney transplant recipients and control groups. We assumed a ratio of 1:1 on age and sex with the nearest neighbor matching method. The propensity score matching was performed using the RStudio v.4.0.2 software.

Based on previous studies, we accepted the seropositivity rate following COVID-19 as 60% for transplant patients and 90% for the general population. Therefore, considering the percentage from the previous studies, we performed power analysis (G*Power software version 3.1; Heinrich-Heine-Universität Düsseldorf, Düsseldorf, Germany) with a power of 90% and an error of 0.05 to determine the minimum sample size for the RT-PCR-positive kidney transplant recipients and control groups. A minimum sample size of 42 was estimated for each group.

## 3. Results

### 3.1. Demographic, clinical and laboratory data

We recruited a total of 91 kidney transplant recipients. Of them, 46 had RT-PCR-confirmed COVID-19, whereas 45 did not have a history of COVID-19 (Table 1). Demographic, clinical, and laboratory data of the transplant recipients grouped according to their COVID-19 status are shown in Table 1. Both groups were similar regarding age and sex. The etiology of CKD, donor type, and posttransplant duration were also similar between the two groups. However, patients with COVID-19 had a higher BMI, and COVID-19 history in a household member was more common among them. Other parameters were similar between the two groups.

The majority (95.6%) of the patients with COVID-19 were symptomatic, and according to computed tomography of the thorax, 30 patients (65.2%) had findings compatible with COVID-19. There was no need for hospitalization in 12 patients (26.1%); the remaining 34 patients (73.9%) were hospitalized, 16 patients (34.8%) needed oxygen, and three patients (6.5%) were followed up in the intensive care unit. Two patients (4.3%) needed intubation. Except for one patient who died on the 26th day of the infection, all patients recovered from COVID-19. The hospitalization duration was 11.7 ± 7.9 days (median: 9 days, range: from 3 to 38 days). According to the COVID-19 severity index, two patients (4.3%) were asymptomatic or presymptomatic, 15 (32.6%) had a mild illness, 16 (34.8%) had a moderate illness, 10 (21.7%) had a severe illness, and three (6.5%) had a critical illness.

### 3.2. Seropositivity

The seropositivity rates and IgG levels among kidney transplant recipients and controls stratified by the COVID-19 status are shown in Table 2. Among the subjects with COVID-19 history, the SARS-Cov-2 IgG positivity rate (69.6% vs. 78.3%) and IgG level (3.28 vs. 4.59 S/C) of kidney transplant recipients were similar to those of the control group. The frequency of COVID-19 related symptoms was more common among kidney transplant recipients than that of the controls; however, the frequency of pulmonary involvement assessed by computed tomography was similar between the two groups. There was no statistically significant difference between the kidney transplant recipients and controls in terms of the duration between RT-PCR and antibody testing (Table 2).

Among the subjects without COVID-19 history, three kidney transplant recipients had positive IgG antibodies, and two of them had a history of COVID-19 in a household member. The SARS-Cov-2 IgG antibody positivity rate (6.7% vs. 6.7%) and IgG level (0.03 vs. 0.03 S/C) of kidney transplant recipients were similar to that of the control group (Table 2).

### 3.3. Predictors of antibody positivity

We compared the demographic, clinical, laboratory, and treatment-related data of the transplant patients who developed antibodies with those who did not (Table 3). The median duration between RT-PCR and antibody testing was shorter (37.5 days [IQR: 20.5–57.8] vs. 82.5 days [IQR: 52.3–105.0], p = 0.01) in patients who had SARS-Cov-2 IgG antibodies compared to that of the patients who did not have IgG antibodies. There were no statistically significant differences between the two groups regarding demographic, clinical, and laboratory parameters. Additionally, the cessation rate of different immunosuppressive drugs was also similar between the two groups.

As an additional analysis, we looked at the correlation between different laboratory parameters (peak CRP, peak ferritin, fibrinogen, peak D-dimer, peak procalcitonin, e-GFR) and the level of SARS-Cov-2 IgG antibodies. There was no significant correlation between those parameters (data not shown).

Finally, we analyzed the correlation between the SARS-Cov-2 IgG antibody levels and the duration between RT-PCR and antibody testing. The antibody level in kidney transplant recipients and controls based on the duration following RT-PCR testing is shown in Figure. Visual examination revealed that kidney transplant recipients who had a longer duration between RT-PCR and antibody testing had lower antibody levels. There was a significant correlation between the antibody levels and the duration between RT-PCR and antibody testing in transplant recipients (r = −0.532, p < 0.001), whereas no statistically significant correlation was found between the two parameters in the controls (r = 0.198, p = 0.186). Additionally, we constructed a multivariate regression model where we used antibody levels as the dependent variable and the study group along with the duration following RT-PCR testing as the independent variables. This analysis showed that the study group was not an independent determinant of antibody levels (data not shown).

## 4. Discussion

We found that kidney transplant recipients developed an antibody response following COVID-19; 69.6% of the patients with COVID-19 history had IgG antibodies and the mean antibody level and the seropositivity rate were similar to that of the control group. To the best of our knowledge, the largest report about antibody response in kidney transplant recipients is from Azzi et al. [9]. In this report, the researchers examined 69 kidney transplant recipients who had an RT-PCR-confirmed COVID-19 diagnosis, and 55 (80.0%) of them had a positive antibody response. The authors used the same test as ours to measure the antibody levels after a median of 44 days following RT-PCR positivity. Hartzell et al. examined anti-SARS-Cov2 IgG antibodies in 16 kidney transplant recipients following a mean of 16.1 days of RT-PCR testing and reported the antibody positivity rate as 60.0% and 63.6%, depending on immunosuppressive drug use [17]. Burack et al. examined 39 kidney transplant recipients and found an antibody positivity rate of 41.0% [7].

We did not identify any specific risk factors for lack of seroconversion following COVID-19. However, we noted a trend toward lower antibody levels in patients who had a longer postinfection duration. A similar observation was reported by Benotmane et al. [18] as they examined 29 kidney transplant recipients hospitalized for COVID-19 and measured antibody levels up to six months after COVID-19. During the follow-up, 20.7% of the patients became seronegative. A considerable IgG reduction was observed in patients treated with calcineurin inhibitors and steroids. No statistically significant difference was found regarding disease severity.

In kidney transplant recipients who did not have positive RT-PCR testing, we found a similar SARS-CoV-2 IgG antibody positivity rate to that in our controls. In line with our results, a recent study reported that the seroprevalence rate was 6.6% in asymptomatic people [19]. In another study, SARS-CoV-2 seroprevalence in healthy blood donors was reported as 3% [20].

Several studies have reported that kidney transplant recipients with COVID-19 have a high mortality rate as high as 40% [1–6]. It has been shown that increasing age and the presence of comorbid diseases are associated with an increased risk of mortality [21,22]. In our study, all kidney transplant recipients recovered except one. All the patients followed the infectious control measures rigorously, while most of the infected patients were younger, had low creatinine levels, and did not have many comorbidities. We closely followed up those patients and changed the immunosuppressive therapy as soon as we were aware of the COVID-19 infection. These factors might be responsible for the low mortality in our study.

Our study had some limitations. We did not formally screen all patients; we might have overlooked some patients who had severe COVID-19 and died. However, it is unlikely that we missed mild cases because most of the patients were in close telephone contact with transplant coordinators during this period. Because of the pandemic, transplantation activity was stopped. Therefore, our cohort consists of long-term transplanted patients. The absence of the patients in the first six months after transplantation when immunosuppression is the strongest might have influenced the results. Another limitation of this study was that the date of infection was defined as the date of PCR positivity, as opposed to the date of symptom onset. Since transplant recipients may exhibit prolonged shedding of the virus, the date of PCR positivity may not always be an accurate estimate of the infection onset. In addition, kidney transplant recipients and controls had different distribution characteristics regarding the duration between COVID-19 and antibody testing. All the controls were tested for antibodies at least four weeks, while no controls were tested beyond 12 weeks postinfection. However, the distribution of the transplant recipients between different IgG testing durations was homogenous. Finally, we did not examine the parameters related to cellular immunity.

In conclusion, kidney transplant recipients seem to have an antibody response similar to that of the general population at the early post-COVID-19 period. However, similar to the general population, there is a tendency toward lower antibody levels with increasing postinfection duration. Therefore, we suggest a caution for humoral immunity in kidney transplant recipients following COVID-19; at least for three months postinfection. The follow-up of antibody levels and booster vaccination might also be warranted.

## Figures and Tables

**Figure:**
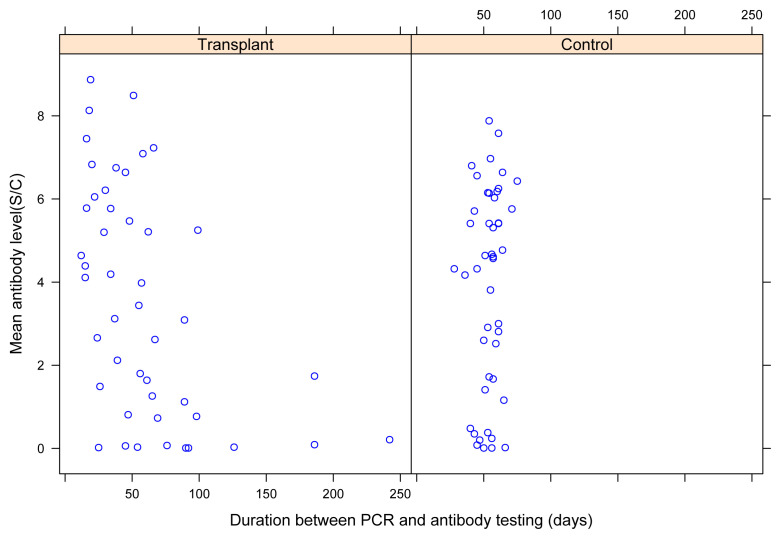
SARS-CoV-2 IgG antibody levels among transplant recipients and controls, according to the duration between RT-PCR and IgG testing.

**Table 1:** Demographic, clinical, and laboratory parameters of the kidney transplant recipients according to their COVID-19 status.

	COVID-19 (+) (n = 46)	COVID-19 (−) (n = 45)	p
Age (years)	46.5 (36.8–55.0)	37.0 (32.0–55.0)	0.194
Male, n (%)	32 (69.6)	27 (60.0)	0.231
Etiology of CKD			
Diabetes mellitus, n (%)	5 (10.9)	3 (6.7)	0.599
Glomerulonephritis, n (%)	8 (17.4)	11 (24.4)	
Others, n (%)	33 (71.7)	31 (68.9)	
Living donor, n (%)	36 (78.3)	35 (77.8)	0.600
Transplantation duration (months)	108.0 (48.0–147.0)	84.0 (24.0–132.0)	0.378
COVID-19 in a household member, n (%)	29 (64.4)	3 (6.8)	<0.001
Comorbidities			
Diabetes, n (%)	13 (28.3)	11 (24.4)	0.431
Hypertension, n (%)	31 (67.4)	26 (57.8)	0.232
BMI (kg/m^2^)	27.4 ± 4.5	24.7 ± 4.2	0.004
Serum creatinine (mg/dL)	1.28 (1.01–1.53)	1.23 (1.02–1.72)	0.778
e-GFR* (mL/min/1.73 m^2^)	64.9 ± 24.1	64.6 ± 31.6	0.955
Baseline immunosuppression			
Steroids, n (%)	46 (100.0)	42 (93.3)	0.117
Calcineurin inhibitor, n (%)	43 (93.5)	43 (95.6)	0.511
Mycophenolic acid derivatives, n (%)	41 (89.1)	35 (77.8)	0.119
m-TOR inhibitors, n (%)	4 (8.7)	4 (8.9)	0.631
Azathioprine, n (%)	3 (6.5)	7 (15.6)	0.149

Values are presented as mean ± SD or as median and IQR.

CKD: chronic kidney disease, BMI: body mass index, m-TOR inhibitors: mechanistic target of rapamycin inhibitors.

* Calculated using the CKD-EPI formula.

**Table 2:** Seropositivity among the kidney transplant recipients and controls stratified by their COVID-19 status.

	COVID-19 (+)		p	COVID-19 (–)		p
	Kidney transplant recipients (n = 46)	Controls (n = 46)		Kidney transplant recipients (n = 45)	Controls (n = 45)	
Age (years)	45.9 ± 12.1	41.3 ± 10.2	0.053	37.0 (32.0–55.0)	37.0 (27.5–53.0)	0.534
Male, n (%)	32 (69.6)	32 (69.6)	0.589	27 (60.0)	27 (60.0)	0.585
Symptoms, n (%)	44 (95.7)	36 (78.3)	0.013	NA	NA	NA
CT result, n (%)	30 (65.2)	30 (65.2)	0.558	NA	NA	NA
IgG positivity rate, n (%)	32 (69.6)	36 (78.3)	0.238	3 (6.7)	3 (6.7)	0.662
IgG level (S/C)	3.28 (0.80–5.85)	4.59 (1.61–6.06)	0.499	0.03 (0.02–0.05)	0.03 (0.02–0.09)	0.997
Days following RT-PCR test	49.5 (25.8–70.6)	55.0 (49.3–61.0)	0.392	NA	NA	NA

CT: computed tomography, Ig: immunoglobulin, RT-PCR: reverse transcriptase-polymerase chain reaction.

Values are presented as mean ± SD or as median and IQR.

**Table 3:** Comparison of the transplant recipients with and without IgG positivity following RT-PCR-confirmed COVID-19.

	IgG-positive (n = 32)	IgG-negative (n = 14)	p
Age (years)	47.5 ± 11.1	42.2 ± 13.8	0.172
Male, n (%)	23 (71.9)	9 (64.3)	0.427
Etiology of CKD			
Diabetes mellitus, n (%)	4 (12.5)	1 (7.1)	0.364
Glomerulonephritis, n (%)	7 (21.9)	1 (7.1)	
Others, n (%)	21 (65.6)	12 (85.7)	
Living donor, n (%)	24 (75.0)	11 (84.6)	0.392
Transplantation duration (months)	108.9 ± 61.9	88.0 ± 78.8	0.337
Comorbidities			
Diabetes, n (%)	10 (31.3)	3 (21.4)	0.381
Hypertension, n (%)	22 (68.8)	9 (64.3)	0.511
Coronary artery disease, n (%)	7 (21.9)	5 (35.7)	0.264
Chronic obstructive pulmonary disease, n (%)	2 (6.3)	1 (7.1)	0.673
BMI (kg/m^2^)	27.6 ± 4.3	26.9 ± 5.1	0.529
Serum creatinine (mg/dL)	1.33 (1.03–1.48)	1.15 (0.87–1.83)	0.867
e-GFR* (ml/min/1.73 m^2^)	63.5 ± 19.0	68.3 ± 33.6	0.537
Disease severity index			
Asymptomatic - moderate, n (%)	21 (66.0)	12 (85.0)	0.286
Severe - critical, n (%)	11 (34.0)	2 (15.0)	
Days following RT-PCR test	37.5 (20.5–57.8)	82.5 (52.3–105.0)	0.001
Lowest white blood cell count, (/mm^3^)	4925.7 ± 1748.0	4596.8 ± 1667.2	0.582
Lowest lymphocytes count, (/mm^3^)	791.0 ± 603.0	665.4 ± 314.7	0.875
Peak CRP, (mg/dL)	75.8 ± 66.5	74.7 ± 58.0	0.813
Peak ferritin, (ng/mL)	625.0 (249.5–1468.3)	670.1 (236.0–1245.0)	0.839
Fibrinogen, (mg/dL)	447.8 (4.98–649.8)	329.1 (4.76–515.5)	0.439
Peak D-dimer, (μg/mL)	1.03 (0.36–2.94)	0.91 (0.66–2.78)	0.868
Peak procalcitonin, (ng/mL)	1.72 (0.92–127.0)	92.5 (1.13–191.3)	0.146
Peak uric acid, (mg/dL)	8.1 ± 2.1	7.7 ± 2.3	0.622
Stopping MPA, n (%)	17 (53.1)	7 (50.0)	0.549
Stopping CNI, n (%)	3 (9.4)	0 (0.0)	NA
Stopping MPA or CNI, n (%)	18 (56.3)	7 (50.0)	0.471

CKD: chronic kidney disease, BMI: body mass index, RT-PCR: reverse transcriptase-polymerase chain reaction, MPA: mycophenolic acid derivatives, CNI: calcineurin inhibitor.

* Calculated using the CKD-EPI formula.

Values are presented as mean ± SD or as median and IQR.
